# Variable temperatures across different stages have novel effects on behavioral response and population viability in a host-feeding parasitoid

**DOI:** 10.1038/s41598-018-38087-0

**Published:** 2019-02-18

**Authors:** Yi-Bo Zhang, Gui-Fen Zhang, Wan-Xue Liu, Fang-Hao Wan

**Affiliations:** 1 State Key Laboratory for Biology of Plant Diseases and Insect Pests, Institute of Plant Protection, Chinese Academy of Agricultural Sciences, Beijing, China; 2 Scientific Observing and Experimental Station of Crop Pests in Guilin, Ministry of Agriculture, Guilin, China

## Abstract

Parasitoids are insects (usually wasps or flies) that lay eggs within or on other insects (their hosts). Host-feeding parasitoids lay eggs to parasitize the host and feed directly on the host for nourishment. Temperature is the most critical factor affecting insect behavioral responses. Few studies have focused on the impacts of variable temperatures across different life stages on the behaviors of host-feeding parasitoids. This study investigated the effects of temperature experienced during the preadult and adult stages on the life history traits and life table parameters of females of a host-feeding parasitoid, *Eretmocerus hayati*. Our results show that the temperatures experienced during the preadult and adult stages significantly change life history traits (immature development, adult longevity, host feeding and fecundity). Increasing the preadult temperature resulted in shorter development times for immature stages of the parasitoid, and decreasing the temperature during the adult stage increased reproduction and longevity. Most importantly, we found that host-feeding events changed with temperature rather than life stage. The daily host-feeding ability of the parasitoid increased with increasing temperature at all temperatures except the stress temperature (34 °C). Furthermore, switching temperatures at the immature stage and adult stage can increase the values of life table parameters, with the highest intrinsic rate of increase (*r*) occurring in the 30/26 °C treatment. This study provides new insight into the mass rearing of parasitic natural enemies.

## Introduction

Parasitoids are insects (wasps or flies) in which adult females forage for hosts, usually other insects, and deposit their eggs in, on, or near these hosts^1,2^. Since the foraging behavior of females is directly linked to their reproductive success, parasitoids are interesting biological models for addressing questions in behavioral ecology^1^. Temperature influences all levels of biological organization^3–5^. With an increase in temperature, the higher kinetic energy of biochemical reactions speeds up the rate of metabolic processes, which subsequently affects the physiology and behavior of individual ectotherms^3–5^. Because parasitoids are typical ectotherms, their behaviors are closely linked to the environmental temperature^5–8^. Previous studies have found that temperature can indirectly or directly affect behavioral decisions through its influence on many fitness traits, such as initial egg load^9,10^, patch residence time^9–12^, oviposition rate^12^, lifetime fecundity^9,10,13^, and the learning reliability of parasitoids^12^. So far, a third of parasitoid species (almost 100 000 species) have been found to be host-feeding parasitoids, indicating that they can suppress the populations of their hosts as a result of parasitization and/or by feeding on their hosts when they are in the adult stage^14^. However, to date, most studies on host-feeding parasitoids have mainly focused on their behavioral and physiological ecology^15–17^, and the impacts of changing temperatures on the varying life stages of long-feeding parasitoids have received comparatively little attention^18,19^.

Parasitoids are important in the natural control of herbivorous insect populations and are often used as biological control agents in agriculture^1^. How to maximize their lifetime fitness is a pivotal question, especially in terms of optimizing their reproductive ability to increase the efficacy of biological control^20^. Many previous studies have compared the life table parameters of parasitoids at different constant temperatures to identify the best intrinsic rate of increase (*r*)^18,19,21–25^. The developmental rates of larval parasitoids usually increase monotonically with temperature, showing a monotonic left-skewed pattern, while reproduction/fecundity shows a symmetrical unimodal pattern^3,22–28^. This suggests that larval development and adult fecundity respond differently to temperature increases. Furthermore, as one of the most important population parameters in life table studies, *r* is strongly influenced by the duration of time from birth to the start of reproduction and, to a lesser extent, by survival and reproduction rates^22,28^^,^. Hence, it is not reasonable to choose the best *r* value by directly comparing them among different constant temperatures over an entire generation, as the net effect of temperature on parasitoid fitness should depend on its relative influence on the immature and adult stages^22–29^. However, surprisingly few studies have considered thermal variation across different life stages^9,10^.

*Eretmocerus hayati* Zolnerowich & Rose (Hymenoptera: Aphelinidae) is a host-feeding primary parasitoid of the invasive agricultural pest *Bemisia tabaci* (commonly known as whitefly, Hemiptera: Aleyrodidae). An adult *E. hayati* parasitizes and feeds directly on its hosts^16,30,31^. The main purpose of this study was to explore how variable temperatures experienced during the preadult and adult stages modify the life-history parameters of *E. hayati*. The immature development time, lifetime feeding, lifetime fecundity, and adult longevity of *E. hayati* reared on the *B. tabaci* cryptic species MED were examined at different combinations of temperatures experienced during the preadult and adult stages, and the biological efficacy and life table parameters under the different treatments were analyzed. We speculate that the immature development time and adult longevity decrease with increasing temperature experienced at preadult stages, while lifetime fecundity and host-feeding events are influenced by the temperatures experienced at the preadult and adult stages. Furthermore, we hypothesize that the life table parameters can change in accordance with variable temperature combinations. In other words, increasing the preadult temperature could shorten the developmental time, and variation in the adult temperature could change lifetime fecundity, which could together maximize *r*.

## Results

### Immature development time

The duration of each immature developmental stage (egg + larva, pupa, and all immature stages) significantly decreased with increasing preadult temperature (egg + larva: *F*_3, 151_ = 313.87, *P* < 0.0001; pupa: *F*_3, 151_ = 250.68, *P* < 0.0001, all immature stages: *F*_3, 151_ = 591.59, *P* < 0.0001, Table 1). Parasitoids showed the fastest rate of development at 34 °C and the slowest at 22 °C.

**Table 1:** Developmental time (mean ± se) of *Eretmocerus hayati* reared on *Bemisia tabaci* at four rearing temperatures.

Temperature (°C)	Duration of developmental stage (d)			
	Egg + larva	Pupa	All immature stages	Sample size
22	11.7 ± 0.1a	7.1 ± 0.2 a	18.8 ± 0.2 a	38
26	9.2 ± 0.1 b	4.9 ± 0.1 b	14.1 ± 0.2 b	36
30	8.4 ± 0.1 c	3.4 ± 0.1 c	11.8 ± 0.1 c	40
34	7.8 ± 0.1 d	3.2 ± 0.1 c	10.9 ± 0.1 d	38

Means in columns followed by different lowercase letters indicate significant differences among temperatures.

### Female longevity

**Figure 1:**
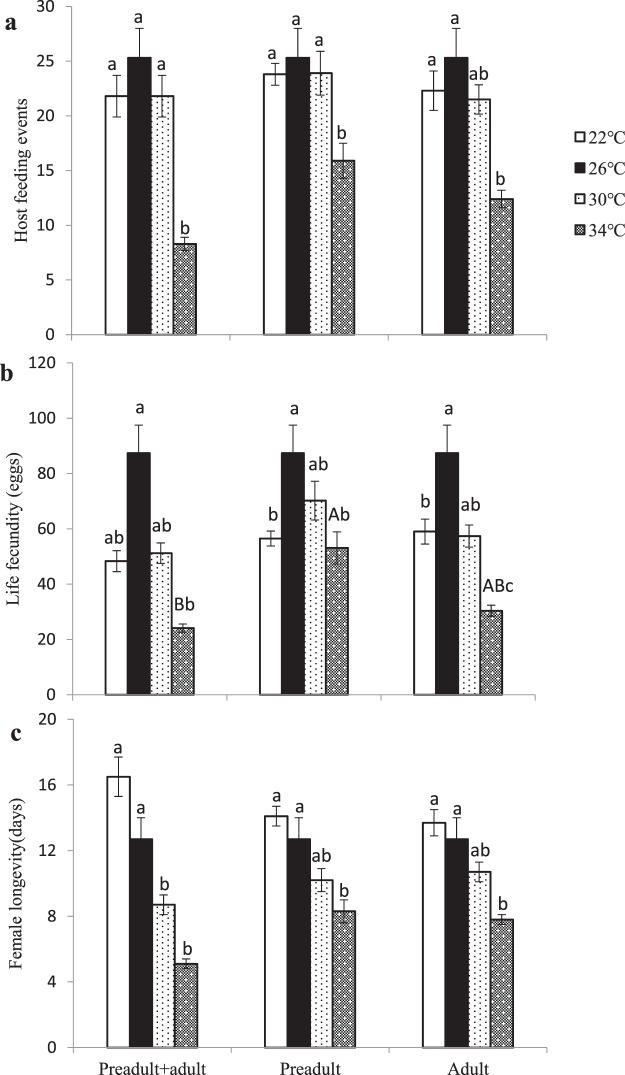
The effects of variable temperatures on the life history traits ((**a**) host feeding, (**b**) lifetime fecundity, (**c**) female longevity) of *Eretmocerus hayati* across different life stages. Bars topped by different capital letters at the same temperature indicate a significant difference among developmental stages; bars topped by different lowercase letters during the same developmental stage indicate a significant difference among temperatures.

Temperature had a significant effect on female longevity (*F*_3, 232_ = 47.98, *P* < 0.0001, Table 2, Fig. 1) but had no impact on developmental stage (*F*_2, 232_ = 0.56, *P* = 0.5712). There was, however, a significant interaction between female longevity and developmental stage (*F*_6, 232_ = 2.75, *P* = 0.0134). When temperatures were changed at the preadult and adult stages simultaneously, the female longevity at 22 °C and 26 °C was significantly longer than that at 30 °C and 34 °C (all Tukey-Kramer tests: *P* < 0.05). However, when temperatures were changed at the preadult stage alone or at the adult stage alone, the longest longevity occurred at 22 °C and 26 °C, intermediate longevity occurred at 30 °C, and the shortest longevity occurred at 34 °C (all Tukey-Kramer tests: *P* < 0.05). When the temperature was fixed, adult longevity did not significantly differ among the developmental stages (all Tukey-Kramer tests: *P* > 0.05).

**Table 2:** Two-way ANOVA results for host feeding, fecundity, adult longevity, daily host feeding and daily fecundity in *Eretmocerus hayati*.

Source	DF	*SS*	Mean Square	*F* value	*P* value
**Host feeding**					
Stage	2	348.18	174.09	2.62	0.0754
Temperature	3	5916.68	1972.22	29.63	<0.0001
Stage*Temperature	6	335.34	55.89	0.84	0.5405
Model	11	6454.04	586.73	8.81	<0.0001
Error	221	14712.19	66.57		
Corrected Total	232	21166.24			
**Fecundity**					
Stage	2	7699.15	3849.57	5.51	0.0046
Temperature	3	77467.19	25822.39	36.94	<0.0001
Stage*Temperature	6	6245.89	1040.98	1.49	0.183
Model	11	89977.91	8179.81	11.7	<0.0001
Error	221	154506.6	699.12		
Corrected Total	232	244484.5			
**Female longevity**					
Stage	2	15.64	7.82	0.56	0.5712
Temperature	3	2005.11	668.37	47.98	<0.0001
Stage*Temperature	6	230.11	38.35	2.75	0.0134
Model	11	2210.1	200.91	14.42	<0.0001
Error	221	3078.68	13.93		
Corrected Total	232	5288.78			
**Daily host feeding**					
Stage	2	1.38	0.69	5.83	0.0034
Temperature	3	18.86	6.28	52.99	<0.0001
Stage*Temperature	6	4.07	0.67	5.72	<0.0001
Model	11	24.12	2.19	18.48	<0.0001
Error	221	26.21	0.11		
Corrected Total	232	50.33			
**Daily fecundity**					
Stage	2	39.38	19.69	13.01	<0.0001
Temperature	3	303.16	101.05	66.76	<0.0001
Stage*Temperature	6	55.79	9.29	6.14	<0.0001
Model	11	396.41	36.03	23.81	<0.0001
Error	221	334.54	1.51		
Corrected Total	232	730.95

### Host feeding and lifetime fecundity

Developmental stage and temperature had significant effects on host feeding (developmental stage: *F*_2, 232_ = 2.62, *P* = 0.0754; temperature: *F*_3, 232_ = 29.63, *P* < 0.0001, Table 2, Fig. 1) and lifetime fecundity (developmental stage: *F*_2, 232_ = 5.51, *P* = 0.0046; temperature: *F*_3, 232_ = 36.94, *P* < 0.0001, Table 2, Fig. 1). There was no significant interaction between host feeding and lifetime fecundity. When the developmental stage was fixed, the greatest host feeding and lifetime fecundity values were attained at 26 °C, intermediate values were found at 22 °C and 30 °C, and the smallest values were found at 34 °C (all Tukey-Kramer tests: *P* < 0.05). Conversely, no significant difference was found in host feeding or lifetime fecundity among the different developmental stages when temperature was fixed (all Tukey-Kramer tests: *P* > 0.05) with the exception of lifetime fecundity when the temperature was 34 °C (Tukey-Kramer test: *P* < 0.05).

### Daily host feeding and daily fecundity

**Figure 2:**
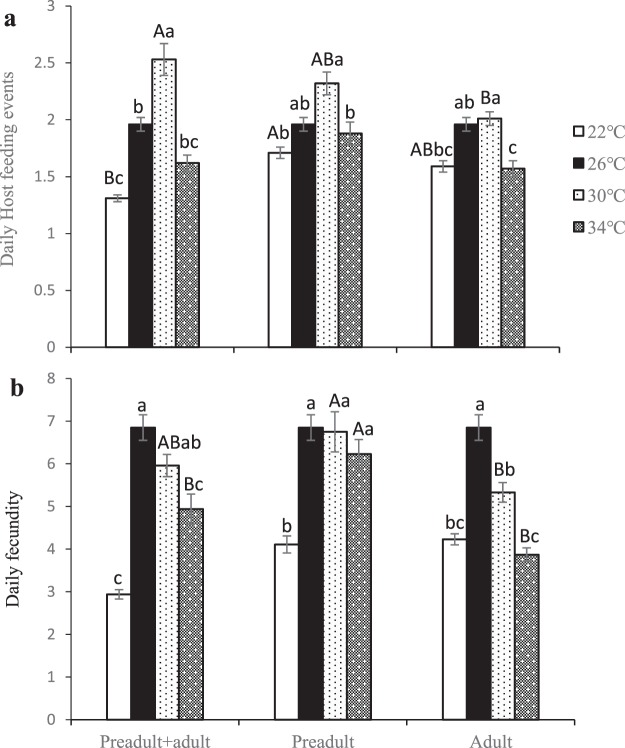
The effects of variable temperatures on (**a**) daily host feeding and (**b**) daily fecundity in *Eretmocerus hayati* across different stages. Bars topped by different capital letters at the same temperature indicate a significant difference among developmental stages; bars topped by different lowercase letters during the same developmental stage indicate a significant difference among temperatures.

Developmental stage and temperature had significant effects on daily host feeding (developmental stage: *F*_2, 232_ = 5.83, *P* = 0.0034; temperature: *F*_3, 232_ = 52.99, *P* < 0.0001, Table 2, Fig. 2) and daily fecundity (developmental stage: *F*_2, 232_ = 13.01, *P* < 0.0001; temperature: *F*_3, 232_ = 66.76, *P* < 0.0001, Table 2, Fig. 2), and there were significant interactions between these variables (host feeding: *F*_6, 232_ = 5.72, *P* < 0.0001; fecundity: *F*_6, 232_ = 23.81, *P* < 0.0001).

When the developmental stage was fixed, the greatest daily host feeding during three different development stage treatments occurred at 30 °C, intermediate values were found at 22 °C and 26 °C, and the smallest values occurred at 34 °C (all Tukey-Kramer tests: *P* < 0.05). However, when the temperature was fixed at 22 °C, the most daily host feeding occurred during the preadult stage alone, while the lowest amount occurred during both the preadult and adult stages (all Tukey-Kramer tests: *P* < 0.05). When the temperature was fixed at 30 °C, the most daily host feeding occurred during both the preadult and adult stages, while the lowest amount occurred during the adult stage alone (all Tukey-Kramer tests: *P* < 0.05). There were no significant differences among the different development stages when the temperature was fixed at 26 °C and 34 °C (all Tukey-Kramer tests: *P* > 0.05).

When the developmental stage was fixed at both the preadult and adult stages, the greatest daily fecundity occurred at 26 °C, intermediate daily fecundity occurred at 30 °C and 34 °C, and the lowest daily fecundity occurred at 22 °C (all Tukey-Kramer tests: *P* < 0.05). Similarly, when the developmental stage was fixed at the adult stage only, the greatest daily fecundity also occurred at 26 °C, but the lowest occurred at 34 °C (all Tukey-Kramer tests: *P* < 0.05). When the developmental stage was fixed at the preadult stage only, the daily fecundities at 26 °C, 30 °C, and 34 °C were significantly greater than those at 22 °C (all Tukey-Kramer tests: *P* < 0.05), and there were no significant differences among the effects of 26 °C, 30 °C, and 34 °C (all Tukey-Kramer tests: *P* > 0.05). When the temperature was fixed at 30 °C or 34 °C, the greatest effect on daily fecundity was found at the preadult stage only, and the smallest effect on daily fecundity occurred during the adult stage only (all Tukey-Kramer tests: *P* < 0.05). There were no significant differences when the temperature was fixed at 22 °C or 26 °C (all Tukey-Kramer tests: *P* > 0.05).

### Life table and population parameters

When the temperature was altered at both the preadult and adult stages, *r* and finite rate of increase (*λ*) were at their highest at 30 °C/30 °C, intermediate at 26 °C/26 °C, and lowest at 34 °C/34 °C (all *P* < 0.05). However, the net reproductive rate (*R*_0_), mean generation time (*T*), and gross reproductive rate (*GRR*) decreased significantly with increasing temperature (all *P* < 0.05) in all treatments except the 22 °C/22 °C treatment (Table 3).

**Table 3:** Population parameters (*r*, intrinsic rate of increase [d^−1^]; *λ*, finite rate of increase [d^−1^]; *R*_0_, net reproductive rate [offspring individual^−1^]; *T*, mean generation time [d]; and *GRR*, gross reproductive rate [offspring individual^−1^]) of *Eretmocerus hayati* reared on *Bemisia tabaci* at different temperatures.

Temperature (°C)	*r*	*λ*	*R*_0_	*T*	*GRR*
**Both preadult and adult stages**					
22/22	0.1258 ± 0.0074 Bb	1.1341 ± 0.0084 Bb	24.2 ± 4.3 B	25.2 ± 0.4 Aa	48.5 ± 3.5 Bb
26/26^※^	0.1940 ± 0.0103 A(B)	1.2141 ± 0.0125 A(B)	43.6 ± 8.9 A(A)	19.4 ± 0.5 B(B)	120.6 ± 24.4 A(A)
30/30	0.2149 ± 0.0120 Ab	1.2398 ± 0.0149 Aab	25.7 ± 4.4 B	15.0 ± 0.2 Cb	52.7 ± 5.1 B
34/34	0.1830 ± 0.0137 Ab	1.2009 ± 0.0164 Aab	12.0 ± 2.1 Cb	13.5 ± 0.1 Dc	18.1 ± 2.1 Cb
**Preadult stage only**					
22/26	0.1442 ± 0.0076 Cb	1.1551 ± 0.0088 Cb	28.2 ± 4.6 A	23.1 ± 0.3 Ab	45.0 ± 3.9 Cb
30/26	0.2304 ± 0.0131 Aa	1.2593 ± 0.0164 Aa	35.0 ± 6.6 A	15.4 ± 0.2 Cb	61.8 ± 6.1 B
34/26	0.2175 ± 0.0125 ABa	1.2431 ± 0.0155 ABa	26.5 ± 4.9 Aa	15.0 ± 0.4 Cb	74.2 ± 9.3 ABa
**Adult stage only**					
26/22	0.1780 ± 0.0098 ABa	1.1948 ± 0.0116 ABa	29.5 ± 5.1 AB	19.0 ± 0.3 Ac	60.2 ± 5.9 Ba
26/30	0.1875 ± 0.0101 ABb	1.2062 ± 0.0121 ABb	28.7 ± 5.0 AB	17.9 ± 0.3 Ba	52.8 ± 6.2 B
26/34	0.1586 ± 0.0106 Bb	1.1719 ± 0.0124 Bb	15.2 ± 2.6 Bb	17.1 ± 0.2 Ba	23.0 ± 3.4 Cb

Means in columns followed by different capital letters indicate significant differences among temperature treatments in the preadult +adult stage, preadult stage or adult stage. Means in columns followed by different lowercase letters indicate significant differences among temperature combinations (22/22 vs 22/26 vs 26/22, 30/30 vs 30/26 vs 26/30, 34/34 vs 34/26 vs 26/34).

^※^These data were also used in tests comparing population parameters among the different temperature treatments in the preadult stage and/or adult stage. Capital letters in parentheses after means indicate significant differences among the different temperature treatments in the preadult stage.

When the temperature was changed at the preadult stage only, *r* and *λ* were also highest at 30 °C/26 °C, intermediate at 34 °C/26 °C, and lowest at 22 °C/26 °C (all *P* < 0.05; Table 3). However, *R*_0_, *T*, and *GGR* decreased significantly with increasing temperature in all cases except in the 22 °C/26 °C treatment (all others: *P* < 0.05; Table 3).

When the temperature was altered at the adult stage only, *r* and *λ* were also the highest at 26 °C/26 °C, intermediate at 26 °C/30 °C, and lowest at 26 °C/34 °C (all *P* < 0.05; Table 3). However, *R*_0_, *T*, and *GGR* decreased significantly with increasing temperature in all cases except the 26 °C/22 °C treatment (all *P* < 0.05; Table 3).

When the temperature was switched between 22 °C and 26 °C, *r* and *λ* changed significantly (all *P* < 0.05, Table 3), and optimal values were found for the two parameters at 26 °C/22 °C. When the temperature was switched between 30 °C and 26 °C, *r* and *λ* changed significantly (all *P* < 0.05, Table 3), and optimal values were found for the two parameters at 30 °C/26 °C. When the temperature was switched between 34 °C and 26 °C, *r* and *λ* changed significantly (all *P* < 0.05, Table 3), and optimal values were found for the two parameters at 34 °C/26 °C.

## Discussion

All immature stages of *E. hayati* exhibited the same temperature-dependent trend in duration: shorter developmental stages at higher preadult temperatures. This is consistent with other temperature-dependent studies on parasitoids, such as *Encarsia citrina* Craw^33^, *Microplitis manilae* Ashmead^23^, *Trichogramma pretiosum* Riley^32^ and *Campoletis chlorideae* Uchida^22^. It is likely a common rule that the development rate of parasitoids, typically ectothermic insects, increases with temperature because the impacts of increased temperature on insect performance depend on the ambient temperature and its difference from the insects’ optimal temperature. If the ambient temperature is below the optimal temperature, a temperature increase to close to its optimal temperature will improve an insect’s performance. However, if the ambient temperature is close to the optimal temperature, the temperature increase may decrease the insect’s performance.

Similarly, we found that temperature significantly affected the longevity of female adults regardless of the stage of development. When temperature varied at the preadult stage only, our results demonstrated that females that developed at low temperatures and were then exposed to 26 °C had a longer lifespan than those that developed at higher temperatures. This is consistent with the results found for the parasitoid species *Aphidius colemani*^13^ and *Aphidius ervi*^10^ but differed from the findings of Le Lann *et al*.^9^, who found that when females of the wasp *Aphidius rhopalosiphi* developed at low temperatures and were then exposed to 20 °C, they had a higher metabolic rate and subsequently a lower longevity. These contrasting results could be due to differences in their study species, such as the climatic origin of the populations^9,13^ or their reproductive modes^34,35^.

As a typical host-feeding parasitoid, *E. hayati* feeds directly on its host, not only obtaining nutritional benefits but also killing the host^30^. We found that host feeding was mainly affected by temperature rather than developmental stage. The parasitoids underwent the most lifetime host-feeding events at 26 °C and the least at 34 °C. However, the largest amount of daily host feeding occurred at 30 °C, while the smallest amount occurred at 22 °C. In other words, as the temperature increased, the parasitoids became more active at every temperature except 34 °C. There are two possible explanations for this result: (1) The metabolic rate could increase, with resources for energy being utilized at a higher rate. In this situation, parasitoids need to replenish their energy or nutrients by feeding on the host, which could result in an increase in daily host feeding at higher temperatures. (2) As the temperature increases, the use of nutrient substances, especially that of the limited lipid reserves, for body maintenance could also increase. Therefore, the nutrient levels of female parasitoids would decrease, indirectly increasing their tendency to feed on their hosts. Our results explored the host-feeding behavioral response of a host-feeding parasitoid facing thermal variance. However, we did not take the real-time metabolic rate or morphological and physiological traits into consideration, so further research should be systematically conducted in the future.

In the present study, we found that the lifetime fecundity of *E. hayati* depended on thermal variance and showed a concave thermal reaction norm shape. The greatest effect on lifetime fecundity occurred at 26 °C while the smallest occurred at 34 °C regardless of the developmental stage. This implies that 26 °C, as an intermediate temperature, is the optimal temperature for *E. hayati* and that 34 °C would be a stressful temperature for development. When the temperature varied only at the preadult stage, the different temperatures significantly affected the fecundity of *E. hayati*. These results were consistent with those of previous studies on other parasitoids, such as *Leptopilina heterotoma*^36^ and *Aphidius colemani*^13^. Fecundity in parasitoids generally depends on the egg load, and oogenesis usually commences during the pupal stage^37^. Different preadult temperatures could not only change the initial egg load but also affect the capital resources, further resulting in different behavioral responses^13,36^^,^. However, the results contrasted with those found in *Aphidius rhopalosiphi*^9^, showing that when *A. rhopalosiphi* individuals developed at low temperatures (10 °C) and were then exposed to 20 °C, they laid more eggs per patch than those at higher temperatures. Thus far, the reasons for these contradictory results are not clear.

When the temperature was altered in the adult stage only, the effect of temperature on fecundity was similar to that in the treatments mentioned above. This means that variation in temperature during the adult stage significantly altered the fecundity of *E. hayati*. Amat *et al*.^34^ identified two reproductive modes (thelytokous and arrhenotokous) in *Venturia canescens* females, in which fecundity differed in response to variable thermal conditions experienced during the adult stage. The thelytokous mode was not significantly altered by changes in temperature, but fecundity in arrhenotokous individuals significantly decreased with increasing temperature. The differences in the response to variable temperature between reproductive modes may be accounted for by differences in selective pressure in the preferred habitats of the wasps using these two modes^34^.

When temperature was changed at both the preadult and adult stages, the effects on fecundity were similar to those found in many previous studies, such as those found for *Campoletis chlorideae*^22^, *Eretmocerus mundus*^18^, *Encarsia acaudaleyrodis*^19^, *Microplitis manilae*^23^, and *Encarsia inaron*^24^. These results were similar to those found when constant temperatures were varied across the entire generation. Most of these parasitoids were nonhost-feeding parasitoids, in which almost the entire egg load matures at the pupal stage or before emergence. In host-feeding parasitoids, however, the lifetime egg load includes both the initial egg load that matures during the pupal stage and the eggs that mature during the adult stage^38,39^. Host-feeding parasitoids can replenish nutrients for enhanced fecundity and prolonged longevity during the adult stage through host feeding^15–17^. Although *E. mundus* is a host-feeding parasitoid^40^, Zandi-Sohani and Shishehbor^19^ did not focus on its host-feeding behavior. To the best of our knowledge, our study could be the first to investigate the behavioral responses (oviposition and host feeding) of a host-feeding parasitoid in relation to temperature. However, because it is difficult to estimate the life history traits of a host-feeding parasitoid at different temperatures when host-feeding behavior is obstructed, we could not further analyze the effects on the costs and benefits to life history traits by host feeding under different thermal conditions.

All the fitness traits (lifetime fecundity, daily fecundity, host feeding, and daily host feeding) of the parasitoids sharply decreased at 34 °C. This means that 34 °C is a stressful temperature for *E. hayati*. There are two possible explanations for this phenomenon: (1) higher metabolic rates at higher temperatures cost more in terms of energetic substance requirements, and (2) stressful temperatures may alter the patch exploitation behavior of parasitoids, such that they spend more time resting or grooming on the patch and less time on host feeding along with reduced investment in reproduction. Although we did not record these behaviors, they were frequently observed during the study. Similar results have been previously reported. Le Lann *et al*.^9^ stated that females of the parasitic wasp *A. rhopalosiphi* reared at 25 °C spent considerably more time resting and grooming on a patch than females reared at lower temperatures. This again suggests that 25 °C is a stressful temperature for development. Sentis *et al*.^26^ found that the relationship between the host-handling rate of *Coleomegilla maculata lengi* Timberlake (Coleoptera: Coccinellidae) and temperature was hump shaped, suggesting that their handling activity was reduced at extreme temperatures. This has also been confirmed in other studies^41,42^. We proposed that when facing a stressful thermal environment, parasitoids will not only directly consume more energy resources for body maintenance but also reduce activity to preserve their energy resources.

The population parameters, such as *r* and *λ*, showed an almost symmetrical, unimodal pattern as λ = *e* ^*r*^. The greatest *r* and *λ* values were found in the females from the 30 °C/30 °C treatment when temperature was changed at both the preadult and adult stages, while when temperature was only changed at the preadult stage, the greatest *r* and *λ* values were found in the females from the 30 °C/26 °C treatment. Meanwhile, we found that increasing the preadult temperatures (30 °C) shortened the immature developmental times but that the same preadult temperatures (26 °C) prolonged adult longevity and increased fecundity compared to the effects of the 30 °C/30 °C treatment. In other words, by varying the thermal conditions across the different life stages, we decreased the developmental time from birth to reproductive maturity and improved the net fecundity rate, which further affected the reproductive and host suppression efficacy (Fig. 1S). This suggests that switching temperatures across the preadult stages and the adult stage can accelerate the immature developmental rate, prolong adult longevity and enhance fecundity, further achieving better reproductive and host suppression efficacy. These results also reveal that the net effect of temperature on parasitoid fitness should depend on its relative influence on the preadult and adult stages, so it would be unreasonable to identify the optimal *r* value by directly comparing them from different constant temperatures during the entire generation^21–25^. These results may also have implications for the use of parasitic biological control agents. Switching temperatures across different life stages could improve rearing efficiency during the mass production of biological control agents. For example, immature parasitoids may be allowed to develop at a relatively high temperature to shorten the time from egg to emerging adult. Female parasitoids can then be transferred to an environment with a relatively cooler temperature to increase lifetime fecundity and prolong longevity. Naturally, the improvement in the performance of adult parasitoids would have to be weighed against the cost of decreasing the immature developmental time.

In summary, our study demonstrates that the temperatures experienced during the preadult and adult stages in a host-feeding parasitoid significantly change its life history traits (immature development, adult longevity, host feeding and fecundity). Increasing the preadult temperature resulted in shorter development times for immature stages of the parasitoid, and decreasing the temperature in the adult stage promoted reproduction and longevity. Most importantly, we found that host feeding changed with temperature rather than life stage. The daily host-feeding ability of parasitoids increased with increasing temperature at all temperatures except the stress temperature (34 °C). Furthermore, switching temperatures at the immature stage and adult stage can improve the values of life table parameters. This study provides new insight into the mass rearing of parasitic natural enemies.

## Methods

### Whitefly and parasitoid cultures

Both whiteflies and their parasitoids were collected from cotton fields during the summer of 2012 in the Ha-Mi region of Xinjiang Uygur Autonomous Region, northwestern China (E89°8′, N42°53′, 12 m a.s.l). Then, colonies of these insects were constructed at the Department of Biological Invasions (DBI), Institute of Plant Protection, Chinese Academy of Agricultural Sciences, Beijing, China. All plants and insects were maintained in a climate-controlled chamber at 26 ± 1 °C and 70–80% relative humidity (RH) and under a 14 h:10 h L:D photoperiod.

Colonies of *B. tabaci* MED were isolated and maintained on young cotton plants (*Gossypium hirsutum* L.) in a plastic box (20 × 30 × 20 cm). The cotton plants were grown in black turf soil and were used to culture the whitefly colonies when they had four fully opened leaves. The identity of the whitefly species in the colony was checked each month by sequencing the cytochrome oxidase I mtDNA from the whiteflies and then comparing it with the available sequences in the NCBI database.

To generate a laboratory colony of the parasitoid, *E. hayati* was cultured on 2^nd^–3^rd^ instar nymphs of *B. tabaci* collected from the same site as the parasitoids and maintained on young cotton plants in the greenhouse of the DBI.

### Experimental setup

The temperatures applied in the study were divided into preadult temperatures for preadult parasitoids and adult temperatures for adult parasitoids. The temperature regimen consisted of four levels: 22 °C, 26 °C, 30 °C, and 34 °C, reflecting the temperature gradient the whitefly and parasite had experienced in the field based on five years of observations. To prevent potential maternal effects, all females were reared for several generations (50 generations) at the same temperature (26 °C). To explore the effects of variable temperatures across different life stages on the life history parameters, we used variable temperatures in the preadult and adult stages (preadult temperature/adult temperature: 22 °C/22 °C, 30 °C/30 °C, 26 °C/26 °C and 34 °C/34 °C), variable temperatures in the preadult stage only (22 °C/26 °C, 26 °C/26 °C, 30 °C/26 °C and 34 °C/26 °C), and variable temperatures in the adult stage only (26 °C/22 °C, 26 °C/26 °C, 26 °C/30 °C and 26 °C/34 °C). All experiments were conducted in climate-controlled growth chambers (75 ± 5% RH, 14 h:10 h L:D photoperiod).

### Development of immature parasitoids at different rearing temperatures

For the development experiments, clean cotton plants with four fully open leaves were transferred into a cage (20 × 20 × 20 cm) and covered with fine gauze. We then transferred 100–120 adult whiteflies into this cage. The whiteflies were allowed to lay eggs for 24 h before being removed. The cotton leaves and eggs were incubated at 26 °C for 10 d and then checked daily with a binocular microscope until the whiteflies reached the appropriate developmental stage for parasitization. According to previous experiments, the late 2^nd^ to early 3^rd^ instar stage of the host nymph is the appropriate stage^31^. We found that this stage was reached after 15–17 d.

The cotton plants infested with suitable nymphs were moved into a cage (20 × 20 × 20 cm). Five newly emerged and mated *E. hayati* females were then released into the cage and allowed to lay eggs for 2 h. A total of 12 cotton plants were treated in this manner. These plants were randomly divided among the four preadult temperatures (22 °C, 26 °C, 30 °C, and 34 °C).

The parasitized nymphs were randomly collected and numbered to facilitate the precise monitoring of their larval and pupal development times. As the *E. hayati* eggs were laid between the nymph host and cotton plant^30^, it was too difficult to observe the process of egg hatching or to record the egg stage. Therefore, we considered the egg and larval stages together and named it the “egg + larval” stage.

### Life history of adult parasitoids at different oviposition temperatures

For each experimental oviposition temperature, 20 pairs of newly emerged (less than 6 h old) adult parasitoids were isolated on a cotton plant with four leaves and infested with 2^nd^–3^rd^ instar nymphs (40–50 nymphs per plant). The cotton plants were refreshed daily at 18:00 until all the parasitoids had died. The cotton plants with parasitized whiteflies were transferred to gauze cages (20 × 20 × 20 cm) and kept at their respective oviposition temperatures in climate-controlled growth chambers. If a host was parasitized, mycetoma displacement was visible though the cuticle under a microscope. If a host was directly fed upon, the body became flat and desiccated^30^. Fecundity, host feeding, and longevity were recorded after 8 d. Because adult males and females were kept as pairs and only female parasitoids have host suppression capacity (lifetime fecundity and host feeding), fecundity and host feeding records were assigned to the females, and daily host feeding and daily fecundity were further calculated.

### Life table analysis

To predict the effects of variable temperatures across different stages on the population viability of a host-feeding parasitoid, we collected raw data on the survivorship, longevity, and daily fecundity of individual females. We analyzed the life table traits (such as the intrinsic growth rate^28^) according to the age-stage, two-sex life table method using the computer program TWOSEX-MSChart^43–45^. Following Chi and Liu (1985)^43^, we calculated the age-stage-specific survival rate (*s*_*xj*_), where *x* is age and *j* is the stage, as well as the age-specific survival rate (*l*_*x*_), the age-stage-specific fecundity (*f*_*xj*_), the age-specific fecundity (*m*_*x*_), *R*_0_, *r*, *λ*, and *T*. *R*_0_ was calculated as follows:

1 $$R0=\sum _{x=0}^{\infty }{l}_{x}{m}_{x}$$

*r* was estimated using the iterative bisection method and the Euler-Lotka equation with the age indexed from 0^46^:

2 $$\sum _{x=0}^{\infty }{e}^{-r(x+1)}{l}_{x}{m}_{x}=1$$

*λ* and *T* were calculated as follows:

3 $$\lambda ={e}^{r}$$

4 $$T=\frac{\mathrm{ln}(Ro)}{r}$$

### Statistical analysis

The immature development times (including those of the larva, pupa and all immature stages) for *E. hayati* at the four different preadult temperatures were compared with one-way ANOVA complemented with Tukey’s honestly significant difference (HSD) test, as the raw data met the assumptions of normality and homoscedasticity.

A general linear model (GLM, two-way ANOVA) was used to test for differences in life history traits (lifetime fecundity, lifetime host feeding, adult longevity, daily fecundity, and daily host feeding). Temperature (22 °C, 26 °C, 30 °C, and 34 °C) and developmental stage (preadult only, adult only, and preadult + adult) were used as the two factors in the model, as all life history traits met the assumptions of normality and homoscedasticity. Tukey multiple comparisons among least-square means were conducted when any of the factors in the models were found to be significant. All analyses were carried out with SAS software version 9.2.

Bootstrapping^47^ was used to estimate the means, variances, and standard errors of the population parameters. Because bootstrapping uses random resampling, a small number of replicates can generate variable means and standard errors. To generate less variable results, we used 10,000 replicates. We used the Tukey-Kramer procedure^48^ in the TWOSEX-MSChart computer program to identify differences among treatments involving variable temperatures in both the preadult and adult stages, adult stage only, and preadult stage only.

### Informed consent

Informed consent was obtained from all authors included in the study.

### Research involving human participants and/or animals

This article does not contain any studies involving human participants or animals performed by any of the authors.

## Supplementary information

Supplementary information
