# Supplementary material for: Variable temperatures across different stages have novel effects on behavioral response and population viability in a host-feeding parasitoid
Source: Sci Rep. 2019 Feb 18;9:2202. doi: 10.1038/s41598-018-38087-0 (PMC6379379; doi:10.1038/s41598-018-38087-0)
Supplement: Supplementary file 1 — Supplementary information [file 41598_2018_38087_MOESM1_ESM.pdf]

Supplementary information

Title:

Variable temperatures across different stage have novel effects on behavioral response and population viability in a host-feeding parasitoid

Yi-Bo Zhang [1, 2], Gui-Fen Zhang [1], Wan-Xue Liu [1], Fang-Hao Wan [1*]

1. *State Key Laboratory for Biology of Plant Diseases and Insect Pests, Institute of Plant Protection, Chinese Academy of Agricultural Sciences, Beijing, China*

2. *Scientific Observing and Experimental Station of Crop Pests in Guilin, Ministry of Agriculture, Guilin, China*

*Corresponding author

Fang-Hao Wan

E-mail address: wanfanghao@caas.cn

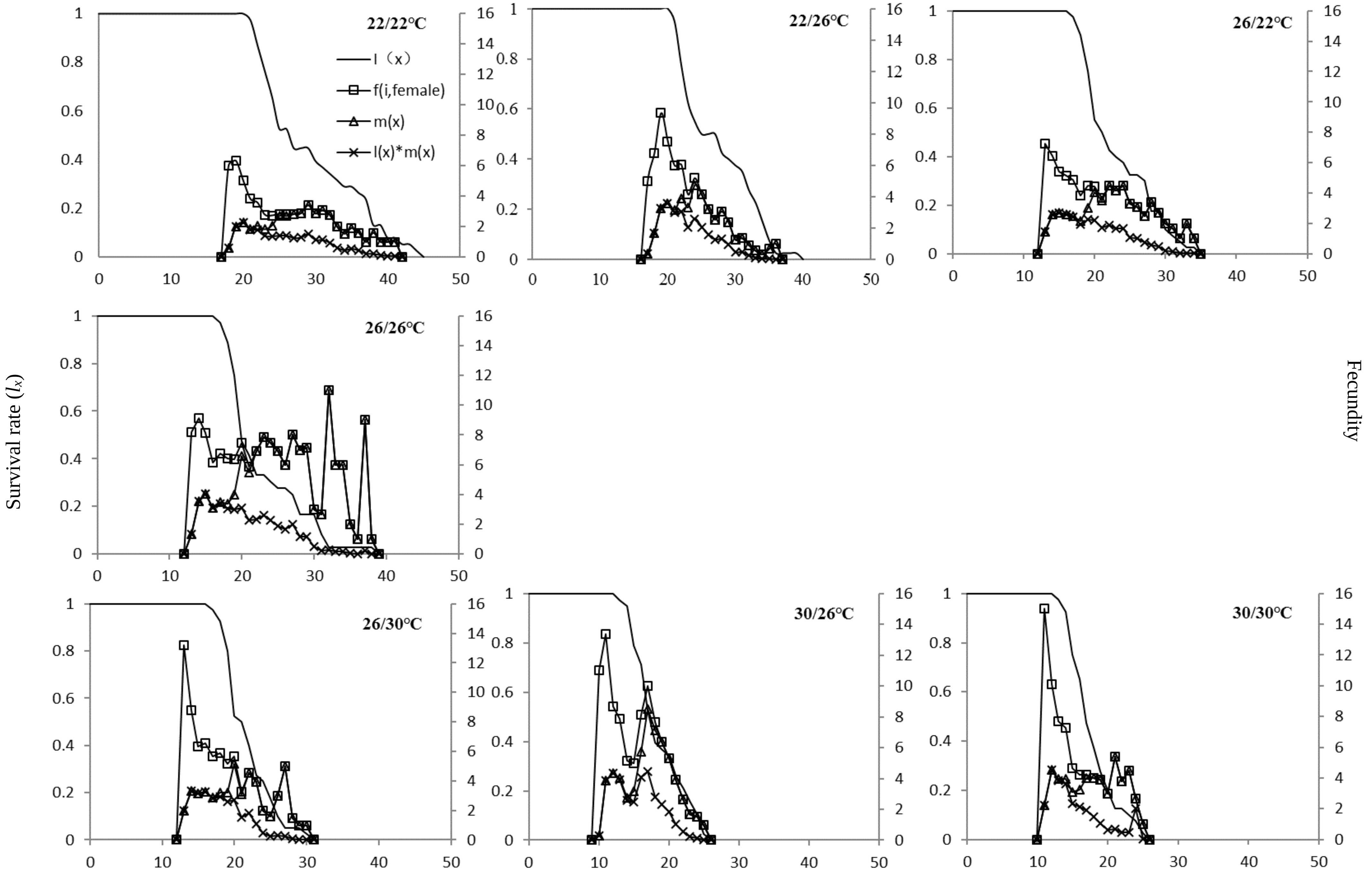
22/22℃
22/26℃
26/22℃
26/26℃
26/30℃
30/26℃
30/30℃
l（x）
f(i,female)
m(x)
l(x)*m(x)
Survival rate (l_x)
Fecundity

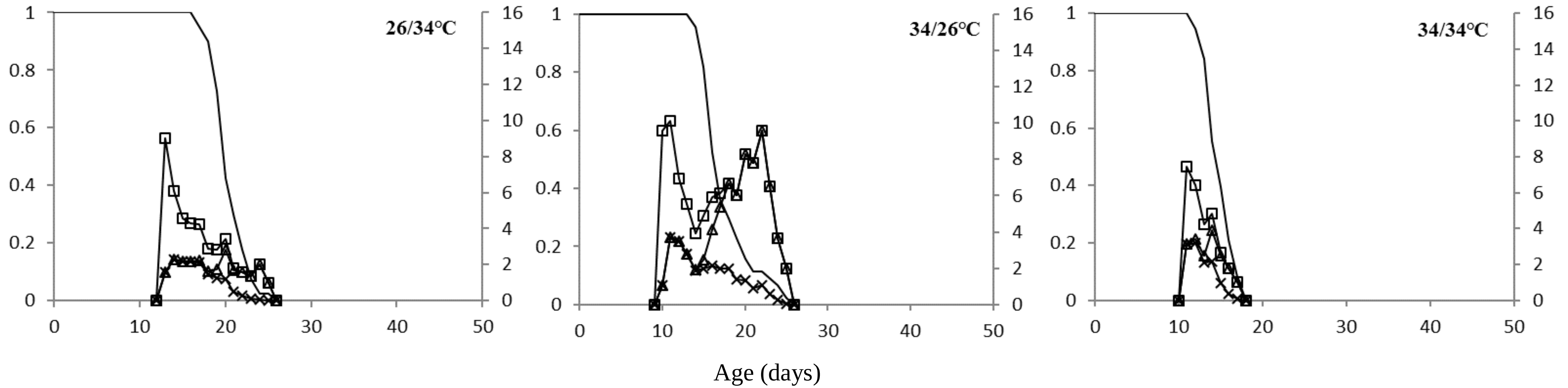

Figure 1S Age-specific survival rate (l_x), female age specific fecundity ($f_{(l,\ female)}$), age-specific fecundity (m_x) and age-specific maternity ($l_x m_x$) of *Eretmocerus hayati* reared on *Bemisia tabaci* nymphs at different temperatures (preadult temperature/adult temperature).
